# Serum levels and renal deposition of C1q complement component and its antibodies reflect disease activity of lupus nephritis

**DOI:** 10.1186/1471-2369-14-63

**Published:** 2013-03-19

**Authors:** Ying Tan, Di Song, Li-hua Wu, Feng Yu, Ming-hui Zhao

**Affiliations:** 1 Renal Division, Department of Medicine, Peking University First Hospital; Institute of Nephrology, Peking University; Key laboratory of Renal Disease, Ministry of Health of China; Key Laboratory of Chronic Kidney Disease Prevention and Treatment, Ministry of Education of China, Beijing 100034, Peoples’ Republic of China; 2 Department of Nephrology, General Hospital of Ningxia Medical University, Ningxia 750004, Peoples’ Republic of China

**Keywords:** Lupus nephritis, Anti-C1q autoantibodies, Serum levels of C1q, C1q depostion

## Abstract

**Background:**

Lupus nephritis is considered to be a principal cause of morbidity and mortality in SLE. Few studies focus on the association between anti-C1q antibodies in circulation and renal C1q deposition in human lupus nephritis. In this study, we detected the serum levels of C1q, presence of anti-C1q antibodies in circulation, renal C1q deposition and further analyzed their associations with clinical and pathological activity in a large cohort of Chinese lupus nephritis patients.

**Methods:**

Sera and renal biopsies from 218 consecutive patients with lupus nephritis with long-term follow up data were studied. Sera were tested for levels of C1q and anti-C1q autoantibodies. Associations of levels of C1q, anti-C1q autoantibodies with renal deposition of C1q, clinical and histopathological data and renal outcome were further investigated.

**Results:**

The levels of serum C1q were significantly lower in lupus nephritis than that in normal controls [33.81 ± 20.36 v.s. 61.97 ± 10.50 μg/ml (*P* < 0.001)]. The prevalence of anti-C1q antibodies, ratios of glomerular and vascular deposition of C1q in patients with lupus nephritis were 42.7% (93/218), 71.6% (156/218) and 86.2% (188/218), respectively. The serum C1q levels and anti-C1q antibodies were associated with SLEDAI scores (*P* < 0.001*, P* = 0.012, respectively), renal total activity indices scores (*P <* 0.001, *P* < 0.001, respectively). Granular positive staining of C1q and IgG by immunofluorescence was co-localized almost completely along the glomerular capillary wall and mesangial areas. Patients with anti-C1q antibodies presented with significantly lower serum C1q levels than those without it (23.82 [0.60, 69.62] μg/ml v.s. 37.36 [0.64, 82.83] μg/ml, *P* < 0.001). The presence of anti-C1q antibodies was associated with the presence of glomerular C1q deposition (*P* < 0.001), but not with the presence of renal vascular C1q deposition (*P* = 0.203).

**Conclusion:**

Anti-C1q autoantibodies were closely associated with serum levels of C1q and glomerular deposition of C1q. Kidney is at least one of the target organs of anti-C1q autoantibodies.

## Backgrounds

Systemic lupus erythematosus (SLE) is an autoimmune disease and is characterized by the production of various autoantibodies. Lupus nephritis is considered to be a principal cause of morbidity and mortality among diseases involving the major organs [1].

C1q is the first component of the classical pathway, which is involved in clearance of immune complexes and apoptotic cells. Interestingly, recent studies showed that C1q could also exert other immuno-regulatory properties, including limiting the differentiation of monocytes into dendritic cells [2] and immune complex–induced interferon-α production in plasmacytoid dendritic cells [3], and participating in neutrophil extracellular trap (NET) degradation [4]. C1q is composed of a collagenous portion and globular heads. Hereditary deficiency of C1q is known to be a risk factor for the development of SLE [5]. Patients with active SLE have lower levels of C1q compared to patients with non-active SLE. Furthermore, patients with lupus nephritis showed lower levels of C1q compared with patients with SLE without nephritis, and the presence of the C1q component in isolated SLE immune complex correlated with the presence of renal disease [6]. Anti-C1q antibodies are prevalent in patients with active lupus nephritis [7] and were thought to be closely associated with renal involvement and predictive for a flare of nephritis [8]. However, the pathogenesis of anti-C1q antibodies involved in lupus nephritis remains unclear.

Animal studies indicated that immune deposition of C1q and anti-C1q antibodies in the kidney was dependent on the presence of glomerular IgG [9-11], while an additional study indicated that anti-C1q autoantibodies deposited in glomeruli but were only pathogenic in combination with glomerular C1q-containing immune complexes [12]. However, the composition of the glomerular and extraglomerular immune complex deposits seems to differ from each other, which indicate that there might be different mechanisms involved in glomerular versus extraglomerular lesions.

There are few published studies that focus on the association between anti-C1q antibodies in circulation and renal C1q deposition in human lupus nephritis. In this work, we detected the serum levels of C1q, presence of anti-C1q antibodies in the circulation, and renal C1q deposition, and further analyzed their associations with clinical and pathological activity in a large cohort of Chinese patients with lupus nephritis.

## Methods

### Patients

Sera and renal biopsies from 218 consecutive patients with renal biopsy-proven lupus nephritis, diagnosed from 2000 to 2008 at Peking University First Hospital were collected on the day of renal biopsy. All the patients fulfilled the 1997 American College of Rheumatology revised criteria for SLE [13].

### Clinical evaluation

Clinical data including gender, presence and degree of fever, malar rash, photosensitivity, oral ulcers, alopecia, arthritis, serositis, neurologic disorder, anemia, leukocytopenia, thrombocytopenia, hematuria, and leukocyturia were noted and analyzed. The clinical disease activity was measured by the Systemic Lupus Erythematosus Disease Activity Index (SLEDAI) [14]. The response to therapy (complete remission, partial remission, relapse, or treatment failure) were the same as in our previous works [15]. All the patients were followed in our outpatient lupus nephritis clinic. The primary end-point was defined as death, and secondary end points were defined as end-stage renal disease (ESRD) or doubling of serum creatinine levels.

### Laboratory assessment

Serum antinuclear antibodies (ANA) were detected using an indirect immunofluorescence assay (EUROIMMUN, Lübeck, Germany) and anti-double-stranded DNA antibodies were detected using a Crithidia luciliae indirect immunofluorescence test (EUROIMMUN, Lübeck, Germany). Anti-extractable nuclear antigen (ENA) antibodies, including anti-Sm, anti-SSA, anti-SSB and anti-RNP antibodies, were detected using immunodotting assays (EUROIMMUN, Lübeck, Germany). Serum C3 was determined using a rate nephelometry assay (Beckman-Coulter, IMMAGE, USA; normal range > 0.85 g/L).

### Renal histopathology

All the renal biopsy specimens were examined by light microscopy, direct immunofluorescence and electron microscopy techniques. Lupus nephritis was re-classified according to the International Society of Nephrology/Renal Pathology Society (ISN/RPS) 2003 classification system [16].

#### Light microscopy examination

Renal biopsy specimens were fixed in 4.5% buffered formaldehyde. Consecutive serial 3 μm sections were used for histological staining. Stains employed included haematoxylin and eosin (H&E), periodic acid-Schiff, silver methenamine (Meth) and Masson’s trichrome. Pathological parameters including activity indices (AI), and chronicity indices (CI) were assessed by renal pathologists using a previously reported system involving semi-quantitative scoring of specific biopsy features, with mild modification [17]. The AI consists of endocapillary hypercellularity, cellular crescents, karyorrhexis/fibrinoid necrosis, subendothelial hyaline deposits, interstitial inflammation, and leukocyte infiltration, and CI includes glomerular sclerosis, fibrous crescents, tubular atrophy, and interstitial fibrosis.

#### Direct immunofluorescence examination

Fresh frozen renal specimens were stained immediately after the renal biopsy with fluoresceinisothiocyanate-labelled rabbit anti-human immunoglobulin G (IgG), immunoglobulin A (IgA), immunoglobulin M (IgM), C3c, C1q and fibrin antibodies (DAKO A/S, Copenhagen, Denmark). Results were graded from 0 to 4 according to the intensity of fluorescence.

To study immune complexes deposited in renal vessels, immunofluorescence staining was performed using rabbit anti-human IgG, IgA, IgM, C3c, C1q, fibrin (DAKO) and C4d (Abcam) on formalin-fixed paraffin-embedded tissue (4 μm thick). Optimal antibody dilutions were pre-determined. In brief, sections were deparaffinized and rehydrated through a series of xylene and graded alcohols. The sections were then treated with 0.4% pepsin (Zhongshan Golden Bridge Biotechnology, Beijing, China) for 40 min. Digestion was terminated by repeated washings in 0.01 mol/L phosphate buffered saline (PBS), pH 7.4. Sections were immersed into freshly prepared 3% hydrogen peroxide in methanol solution for 10 min at room temperature to quench endogenous peroxidase activity. To block non-specific staining, sections were incubated with 3% BSA in PBS at room temperature for 30 min. The primary antibodies against IgG (diluted 1:5000 in PBS), IgA (1:1000), IgM (1:400), C3c (1:300), C4d (1:400), C1q (1:50) and fibrin (1:1000) were added on each section directly. Antibodies were incubated overnight at 4°C. FITC-labeled goat anti-rabbit IgG (Zhongshan Golden Bridge Biotechnology, Beijing, China; diluted 1:60) was used as a secondary antibody at 37°C for 30 min. Sections were washed with PBS (pH 7.4) between each step (5 min, three times). Finally, sections were stored shortly at 4°C before being examined using an immunofluorescence microscope (Nikon Eclipse 80i, Japan). Sections of renal tissue from patients diagnosed with lupus nephritis were used as positive controls. Samples stained without primary antibodies were used as negative controls.

#### Detection of co-localization of C1q and IgG on glomeruli by immunofluorescence

Five μm sections from frozen renal biopsy tissues were cut and placed onto slides, air-dried for 25 min at room temperature, and fixed in pre-cooled acetone for 10 min at 4°C. After three washes with phosphate buffered saline (PBS), sections were incubated in 3% BSA for 30 min at room temperature. Tetramethylrhodamine isothiocyanate (TRITC) -labeled rabbit anti-human IgG (diluted 1:40; Dako, Copenhagen, Denmark) were incubated 30 min at 37°C. After washing with PBS 3 times for 3 min, mouse anti-human C1q (diluted 1:400; Abcam, Cambridge, UK) was added as primary antibody for 30 min at 37°C. After another 3 × 3 min washes with PBS, FITC-labeled goat anti-mouse IgG (diluted 1:30; Zhongshan Golden Bridge Biotechnology, Beijing, China) was used as the secondary antibody for 30 min at 37°C. After washing with PBS 3 times for 3 min, the sections were air-dried in the dark and mounted with citifluor. Samples stained without primary antibodies were used as negative controls. Sections were stored shortly at 4°C before being scored using a confocal microscope (Olympus Viewer 1000, Japan).

### Serum samples

Sera from patients with lupus nephritis were obtained from peripheral blood at the time of renal biopsy. Sera from 22 healthy controls (gender and age matched) were collected as normal controls. All of the sera were stored at −70°C until use.

Written informed consent was obtained for blood sampling and renal biopsy from each patient. The research was in compliance with the Declaration of Helsinki and approved by the ethics committee of Peking University First Hospital.

### Detection of serum C1q levels using enzyme-linked immunosorbent assay (ELISA)

Detection of serum C1q levels was performed by modifying a previously described method [18]. Anti-human C1q polyclonal antibody from rabbit (Dako, Denmark) was diluted to 1:5000 (1.26 μg/ml) in 0.05 M bicarbonate buffer (pH 9.6) and coated onto the wells of a polystyrene microtitre plate (Nunc, Denmark). All plates were coated with 50 μl of this solution and incubated overnight at 4°C, then washed three times with 0.01 M PBST and blocked with 200 μl PBST containing 1% BSA, 37°C for 1 h. The following incubations were carried out at 37°C for 1 h, 50 μl total volume in each well, and all plates were washed three times with PBST. Standards and diluted serum sample (1:2,000 in PBST) were added to their respective wells. All the samples were tested in duplicate wells. Subsequently, horseradish peroxidase (HRP)-conjugated goat anti-human IgG monoclonal antibody (Abcam) was diluted to 1:500. The reaction was developed with 3,3^′^,5,5^′^-Tetramethylbenzidine (TMB) liquid substrate system and was stopped with 1 M H_2_SO_4_. The results were recorded as the net optical absorbance at 450 nm and 570 nm in an ELISA reader (Bio-Rad 550, Japan). The cutoff value of C1q was set as the mean - 2 SD of healthy blood donors.

### Detection of anti-C1q IgG autoantibodies with ELISA

Anti-C1q IgG autoantibodies were detected using a previously published ELISA method [7]. In brief, purified normal human C1q (Sigma, USA) was diluted at 5 μg/ml in 0.05 M bicarbonate buffer (pH 9.6) and coated onto the wells of one-half of a polystyrene microtitre plate (Costar, USA). The wells in the other half were coated with the same bicarbonate buffer alone as antigen-free wells to measure non-specific binding. The volumes used in each well for this step and for subsequent steps was 100 μl. All incubations were carried out at 37°C for 1 h, and all plates were washed three times with 0.01 M PBS containing PBST. Wells were then blocked with PBST containing 0.01% gelatin. The sera diluted to 1:200 with PBST containing 1% BSA and 0.5 M NaCl, and were added in duplicate to both antigen-coated and antigen-free wells. Each plate included a blank control, negative control and a known positive control. After incubation and washing, the wells were then incubated with 1:5000 diluted horseradish peroxidase (HRP)-conjugated goat anti-human IgG (Zhongshan Biotech, China). The reaction was developed with a 0.1 M citrate phosphate buffer (pH 5.0) containing 0.04% O-phenylenediamine (OPD) and 0.1% H_2_O_2_, then the reaction was stopped with 1 M H_2_SO_4_. The results were recorded as the net optical absorbance (average value of antigen wells minus average value of antigen-free wells) at 490 nm in an ELISA reader (Bio-Rad 550, Japan) and expressed as percentage of the known positive sample. The cutoff value was set as the mean + 2 SD of healthy blood donors.

### Statistical analysis

Statistical software SPSS 13.0 (SPSS, Chicago, IL, USA) was employed for statistical analysis. Quantitative data were expressed as mean ± SD., median with range (minimum, maximum), or number (%). For comparison of clinical and pathological features of patients, Mann–Whitney Test, *X*^2^ test and Spearman’s correlation were used. Survival analysis was performed using the log-rank test. Statistical significance was considered as *P* <0.05.

## Results

### General data of patients with lupus nephritis

The clinical and renal histopathological data of 218 lupus nephritis patients at the time of renal biopsy are listed in Table 1.

**Table 1:** General clinical and renal histopathological profiles of patients with lupus nephritis at renal biopsy

**Number of patients**	**218**	**C3 (mean ± SD) (g/l)**	**0.45 ± 0.23**
Gender (male/female)	33/185	Anti-nuclear antibody (ANA) (+) No.(%)	215 (98.6)
Age (mean ± SD) (years)	32.5 ± 11.3	Anti-double stranded DNA antibody (ds-DNA) (+) No.(%)	153 (70.6)
Follow-up time (median, range) (months)	41 (1,360)	Anti-SSA antibody (+) No.(%)	102 (46.8)
Fever (non-infectious) No.(%)	69 31.7)	Anti-SSB antibody (+) No.(%)	25 (11.5)
Malar rash No.(%)	118 (54.1)	Anti-Smith antibody(Sm) (+) No.(%)	53 (24.3)
Photosensitivity No.(%)	47 (21.6)	Anti-ribonucleoprotein (RNP) antibody (+) No.(%)	66 (30.3)
Oral ulcer No.(%)	64 (29.4)	Anti- cardiolipin antibody (+) No.(%)	16 (7.3)
Alopecia No.(%)	68 (31.2)	SLEDAI (mean ± s.d.)	17.6 ± 5.8
Arthralgia No.(%)	115 (52.8)	Activity indices (AI) score (median, range)	8 (0,19)
Serositis No.(%)	34 (15.6)	Endocapillary hypercellualrity (median, range)	3 (0,-3)
Neurologic disorder No.(%)	14 (6.4)	Cellular crescents (median, range)	0 (0,6)
Anemia No.(%)	147 (67.4)	Karyorrhexis/fibrinoid necrosis (median, range)	0 (0,6)
Leukocytopenia No.(%)	102 (46.8)	Subendothelial hyaline deposits (median, range)	1 (0,3)
Thrombocytopenia No.(%)	64 (29.4)	Interstitial inflammatory cell infiltration (median, range)	1 (0,3)
Hematuria No.(%)	168 (77.1)	Glomerular leukocyte infiltration (median, range)	1 (0,12)
Leukocyturia (non-infection) No.(%)	118 (54.1)	Chronicity indices (CI) score (median, range)	2 (0,10)
Acute renal failure No.(%)	39 (17.9)	Glomerular sclerosis (median, range)	0 (0,3)
Hemoglobin (mean ± SD) (g/l)	100.92 ± 26.36	Fibrous crescents (median, range)	0 (0,3)
Urine protein (median, range) (g/24 hours)	4.36 (0–17)	Tubular atrophy (median, range)	1 (0,3)
Serum creatinine (median, range) (μmol/l)	82.0 (37,1000)	Interstitial fibrosis (median, range)	1 (0,3)

*SSA/SSB*, Sjögren’s syndrome A/Bantigen; *SSB*, Sjögren’s syndrome B antigen; *SLEDAI*, SLE Disease Activity Index.

### Levels of serum C1q in patients with lupus nephritis and their associations with clinical and pathological features

Purified human C1q was used to establish the standard curve. C1q could be detected by the sandwich ELISA at a range of 0 μg/ml to 125 μg/ml. The average levels of serum C1q were 33.81 ± 20.36 (0.60, 82.83) μg/ml in the lupus nephritis group, which were significantly lower than that in normal controls (61.97 ± 10.50 [46.05, 86.34] μg/ml, *P* < 0.001). The cutoff value for the lower limit of detection of C1q was 40.97 μg/ml.

Levels of serum C1q were correlated with age (r = 0.161, *P* = 0.018). There was no significant difference in serum C1q levels between female and male with lupus nephritis (*P* = 0.291). Patients with lower serum C1q levels (<40.97 μg/ml) showed significantly higher levels of SLEDAI (*P* < 0.001), significantly lower levels of C3 (*P* < 0.001), and significantly higher incidence of anti-ds-DNA antibody (*P* = 0.003), hematuria (*P* = 0.021), and anemia (*P* = 0.004; Table 2). The levels of serum C1q in different pathological classes of lupus nephritis were 42.39 (9.61, 78.26) μg/ml in class II, 36.00 (0.92, 82.83) μg/ml in class III, 27.21 (0.60, 77.07) μg/ml in class IV, 51.81(1.19, 81.98) μg/ml in class V, and 37.92 μg/ml in class VI. There were significant differences in serum C1q levels among various pathological classes (*P* < 0.001). The level in class IV was the lowest among the four classes (IV v.s. II, *P* = 0.046; IV v.s. III, *P* = 0.005; IV v.s. V, *P* < 0.001; respectively).

**Table 2:** Associations between presence of anti-C1q autoantibodies, levels of C1q and clinical parameters of patients with lupus nephritis

	**Patients with anti-C1q autoantibodies**	**Patients without anti-C1q autoantibodies**	***P *value**	**Patients with levels of C1q above 40.97 μg/ml**	**Patients with levels of C1q under 40.97 μg/ml**	***P *value**
**Demographic data**						
Number of patients (%)	93/218(42.7%)	125/218(57.3%)		75/218(34.4%)	143/218(65.6%)	
Gender (male/female)	14/79	19/106	NS	12/63	21/122	NS
Age (mean ± SD) (years)	31.27 ± 9.96	33.47 ± 11.21	NS	34.15 ± 10.92	31.69 ± 11.40	NS
**Clinical and laboratory data**						
Fever (non-infectious) No.(%)	31(33.3%)	38(30.4%)	NS	19(25.3%)	50(35.0%)	NS
Malar rash No.(%)	51(54,8%)	67(53,6%)	NS	42(56.0%)	76(53.1%)	NS
Photosensitivity No.(%)	20(21.5%)	27(21.6%)	NS	16(21.3%)	31(21.7%)	NS
Oral ulcer No.(%)	28(30.1%)	38(30.4%)	NS	20(26.7%)	44(30.8%)	NS
Alopecia No.(%)	30(32.3%)	38(30.4%)	NS	26(34.7%)	42(29.4%)	NS
Arthralgia No.(%)	60(64.5%)	55(44.0%)	0.003	36(45.6%)	79(55.2%)	NS
Serositis No.(%)	13(14.0%)	21(16.8%)	NS	10(13.3%)	24(16.8%)	NS
Neurologic disorder No.(%)	7(7.5%)	7(5.6%)	NS	1(1.3%)	13(9.1%)	NS
Anemia No.(%)	73(78.5%)	74(59.2%)	0.003	41(54.7%)	106(74.1%)	0.004
Leukocytopenia No.(%)	49(52.7%)	53(42.4%)	NS	23(30.7%)	79(55.2%)	NS
Thrombocytopenia No.(%)	26(28.0%)	38(30.4%)	NS	19(25.3%)	45(31.5%)	NS
Hematuria No.(%)	79(36.2%)	89(40.8%)	0.017	51(68.0%)	117(81.8%)	0.021
Leukocyturia No.(%)	55(25.2%)	63(28.9)	NS	34(45.3%)	84(58.7%)	NS
Acute kidney injury No.(%)	18(8.3%)	21(9.6%)	NS	13(17.3%)	26(18.2%)	NS
Hemoglobin (mean ± SD.) (g/l)	96.30 ± 25.78	104.36 ± 26.36	0.025	102.89 ± 31.52	99.89 ± 23.27	NS
Urine protein (mean ± SD) (g/24 hours)	4.42 ± 3.05	5.40 ± 3.39	NS	5.64 ± 3.70	4.64 ± 3.00	0.045
C3 (mean ± SD) (g/l)	0.39 ± 0.19	0.50 ± 0.25	<0.001	0.60 ± 0.25	0.37 ± 0.17	<0.001
Anti-nuclear antibody (ANA) (+) No.(%)	92(98.9%)	123(98.4%)	NS	73(97.3%)	142(99.3%)	NS
Anti-double stranded DNA antibody (ds-DNA) (+) No.(%)	71(76.3%)	82(65.6%)	NS	43(57.3%)	110(76.9%)	0.003
Anti-SSA antibody (+) No.(%)	45(48.4%)	57(45.6%)	NS	43(57.3%)	59(41.3%)	NS
Anti-SSB antibody (+) No.(%)	8(8.6%)	17(13.6%)	NS	9(12.0%)	16(11.2%)	NS
Anti-Smith antibody(Sm) (+) No.(%)	26(28.0%)	27(21.6%)	NS	21(28.0%)	32(22.4%)	NS
Anti-ribonucleoprotein (RNP) antibody (+) No.(%)	34(36.7%)	32(25.6%)	NS	26(34.7%)	40(28.0%)	NS
Anti- cardiolipin antibody (+) No.(%)	10(10.8%)	6(4.8%)	NS	2(2.7%)	14(9.8%)	NS
SLEDAI	18.72 ± 5.70	16.74 ± 5.69	0.012	15.47 ± 4.90	18.69 ± 5.89	<0.001

*NS*, no significance.

The lower levels of serum C1q (<40.97 μg/ml) were associated negatively with the renal activity indices score (r = −0.327, *P <* 0.001), endocapillary hypercellularity (r = −0.337, *P <* 0.001), cellular crescents (r = −0.182, *P = 0.007*) fibrinoid necrosis (r = −0.310, *P <* 0.001), subendothelial hyaline deposits (r = −0.312, *P <* 0.001), glomerular leukocyte infiltration (r = −0.246 *P <* 0.001), and associated positively with glomerular sclerosis (r = 0.146, *P* = 0.031). No significant associations were found between other indices (Table 3).

**Table 3:** Associations between presence of anti-C1q autoantibodies, levels of C1q and histopathological parameters of patients with lupus nephritis

	**Patients with anti-C1q autoantibodies**	**Patients without anti-C1q autoantibodies**	**r value(*P *value)**	**Patients with levels of C1q above 40.97 μg/ml**	**Patients with levels of C1q under 40.97 μg/ml**	**r value(*P *value)**
	**Median(range)**	**Median(range)**		**Median(range)**	**Median(range)**	
**Histopathological data**						
Activity Indices score	9(0,19)	7(0,16)	0.238(<0.001)	4(0,19)	9(0,19)	−0.327(<0.001)
Endocapillary hypercellularity	3(0,3)	2(0,3)	0.249(<0.001)	3(0,3)	1(0,3)	−0.337(<0.001)
Cellular crescents	2(0,6)	0(0,6)	0.167(0.013)	2(0,6)	0(0,6)	−0.182(0.007)
Karyorrhexis/fibrinoid necrosis	2(0,6)	0(0,4)	0.175(0.009)	2(0,6)	0(0,2)	−0.310(<0.001)
Subendothelial hyaline deposits	1(0,3)	1(0,3)	0.171(0.012)	1(0,3)	0(0,3)	−0.312(<0.001)
Interstitial inflammation	1(0,3)	1(0,3)	NS	1(0,3)	1(0,3)	NS
Leukocyte infiltration	1(0,3)	1(0,3)	0.159(0.019)	1(0,3)	1(0,3)	−0.246(<0.001)
Chronicity Indices score	2(0,9)	2(0,10)	NS	2(0,10)	2(0,9)	NS
Glomerular sclerosis	0(0,3)	0(0,3)	NS	0(0,3)	0(0,3)	0.146(0.031)
Fibrous crescents	0(0,3)	0(0,3)	NS	0(0,3)	0(0,2)	NS
Tubular atrophy	1(0,3)	1(0,3)	NS	1(0,3)	1(0,3)	NS
Interstitial fibrosis	1(0,3)	1(0,3)	NS	1(0,3)	1(0,3)	NS

*NS*, no significance.

### Prevalence of serum anti-C1q IgG auto-antibodies in patients with lupus nephritis and their associations with clinical and pathological characteristics

The cutoff value for detection of anti-C1q IgG auto-antibodies was 29%. The prevalence of anti-C1q antibodies in patients with lupus nephritis was 42.7% (93/218), which was significantly higher than that in normal controls (0/22). Patients with positive anti-C1q antibodies showed significantly higher SLEDAI (*P* = 0.012), lower levels of C3 (*P* < 0.001) and hemoglobin (*P* = 0.025), compared with those without anti-C1q antibodies (Table 2). Anti-C1q antibodies were also associated with arthralgia (*P* = 0.003), anemia (*P* = 0.003) and hematuria (*P* = 0.017; Table 2). The prevalence of anti-C1q antibodies in patients with type II, III, IV, V, and VI lupus nephritis were 25.0% (2/8), 33.3% (13/39), 55.1% (70/127), 16.3% (7/43), and 100% (1/1), respectively. The prevalence of anti-C1q antibodies in patients with diffuse proliferative renal lesions (class IV) was significantly higher than that in patients with non-diffuse proliferative renal lesions (class II, III, V) (55.1% [70/127] v.s. 36.7% [33/90]; *P* < 0.001). Anti-C1q antibodies were associated with most histopathological parameters of activity indices including activity indices score (*P* < 0.001), endocapillary hypercellularity (*P* < 0.001), cellular crescents (*P* = 0.013), karyorrhexis/fibrinoid necrosis (*P* = 0.009), subendothelial hyaline deposits (*P* = 0.012) and leukocyte infiltration (*P* = 0.019; Table 3).

### Co-localization of C1q and IgG deposited in glomerulis by laser scanning confocal microscopy

**Figure 1:**
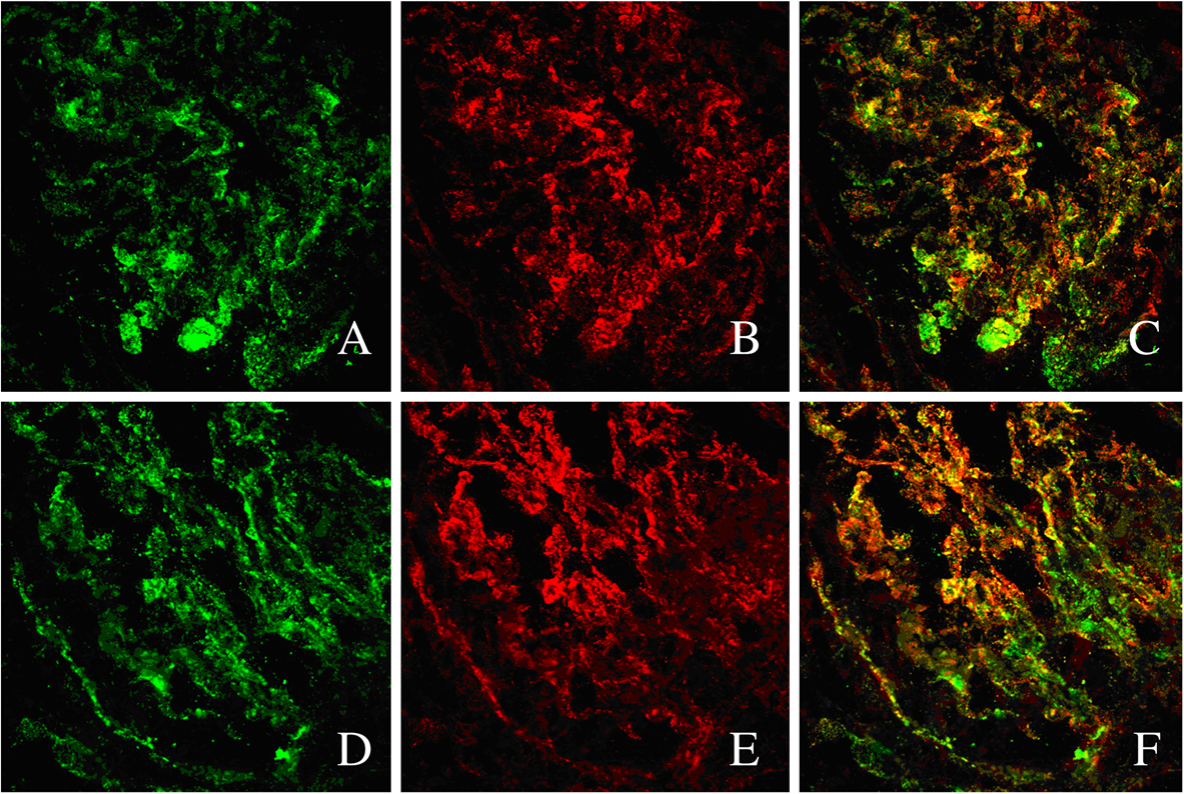
**Co-localization of C1q and IgG staining in glomeruli in lupus nephritis. A**&**D**. Granular positive staining of C1q by immunofluorescence along the glomerular capillary wall and mesangial area in a patient with lupus nephritis. **B**&**E**. Granular positive staining of IgG by immunofluorescence along the glomerular capillary wall and mesangial area in the same section in Figure 1A or D. **C**&**F**. C1q and IgG co-localized completely along the glomerular capillary wall and mesangial area, merged in yellow. (Original magnification × 800).

The prevalence of glomerular and vascular deposition of C1q in patients with lupus nephritis was 71.6% (156/218) and 86.2% (188/218), respectively. Renal biopsies from four patients with lupus nephritis with positive anti-C1q autoantibodies were stained for C1q and IgG. Granular positive staining of C1q and IgG by immunofluorescence was shown along the glomerular capillary wall and mesangial area and was co-localized almost completely in the same areas (Figure 1).

### Correlations among levels of serum C1q, autoantibodies against C1q, glomerular and vascular depositions of C1q in lupus nephritis

**Figure 2:**
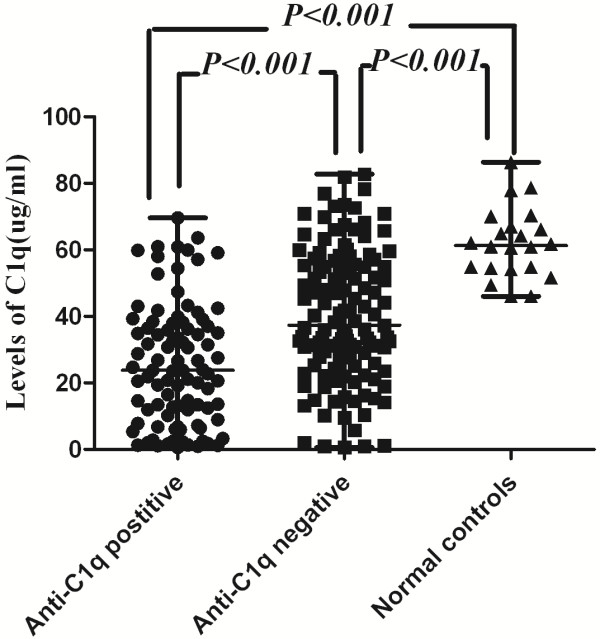
**Comparison of serum C1q levels in patients with lupus nephritis with positive or negative anti-C1q antibodies and normal controls.** The horizontal lines indicated the median value and range.

Patients positive for anti-C1q antibodies presented with significantly lower serum C1q levels than those without anti-C1q antibodies (23.82 [0.60, 69.62] μg/ml vs. 37.36 [0.64, 82.83] μg/ml, *P* < 0.001; Figure 2). However, the serum C1q levels were not associated with the intensity of glomerular or vascular C1q deposition in the kidneys (*P* = 0.469, *P* = 0.198, respectively).

The presence of anti-C1q antibodies were associated positively with the presence of glomerular deposition (87.1% patients with anti-C1q antibodies [81 patients with C1q deposition/93 patients with antibodies] vs. 60.0% patients without anti-C1q antibodies [75 patients with C1q deposition/125 patients without antibodies], *P* < 0.001), but not with the presence of vascular C1q deposition (82.8% patients with anti-C1q antibodies [77 patients with C1q deposition/93 patients with antibodies] vs. 88.8% patients without anti-C1q antibodies [111 patients with C1q deposition/125 patients without antibodies], *P* = 0.203). The intensity of glomerular deposition was not associated with the intensity of vascular deposition (r = −0.081, *P* = 0.239).

### Associations between levels of serum C1q, autoantibodies against C1q, glomerular, vascular deposition of C1q and renal outcomes

**Figure 3:**
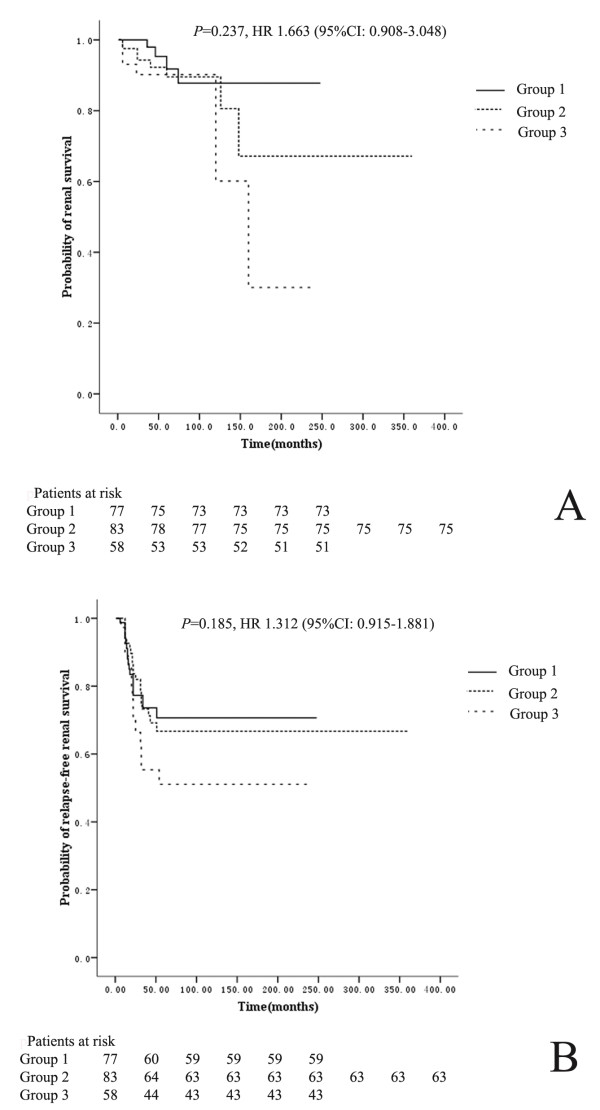
**Comparison of renal outcomes and relapse-free renal survival among patients with different levels of C1q. A**. Comparison of renal outcomes among patients with different levels of C1q; **B**. Comparison of relapse-free renal survival among patients with different levels of C1q. Group 1.patients with “normal C1q” (> 40 μg/ml); Group 2. patients with “low C1q” (20–40 μg/ml); Group 3. patients with “very low C1q” (<20 μg/ml).

Levels of serum C1q, autoantibodies against C1q, or glomerular or vascular deposition of C1q were found to not be risk factors for long-term renal outcomes in lupus nephritis using the log-rank test for univariate survival analysis of renal prognosis (*P* = 0.200, *P* = 0.301, *P* = 0.548, *P* = 0.697, respectively). Patients were divided into three groups according to C1q level (patients with “normal C1q” (> 40 μg/ml), “low levels of C1q” (20–40 μg/ml) and “very low levels of C1q” (<20 μg/ml)). No significant difference between the three groups was found regarding long-term renal survival and relapse-free renal survival (*P* = 0.237, *P* = 0.185, respectively) (Figure 3).

## Discussion

The kidney is one of the most commonly involved organs in SLE. C1q and its autoantibodies have been considered to be associated closely with SLE and lupus nephritis. Controversies still exist in the organ specification of anti-C1q autoantibodies. Recently, Yasuhiro et al. indicated that anti-C1q antibodies were associated with SLE global activity but not specifically with active lupus nephritis [19]. With a large lupus nephritis cohort in our center, we first demonstrated associations among levels of C1q, presence of anti-C1q antibodies, renal C1q deposition and their correlations with clinical and pathological disease activity of lupus nephritis, which might shed a light on the roles of C1q and it autoantibodies in lupus nephritis.

Previous studies have demonstrated a negative correlation between anti-C1q autoantibodies and serum levels of C1q in patients with lupus nephritis. Levels of C1q were significantly lower in patients with lupus nephritis and in active patients [20]. Animal studies indicated that the severity of lupus nephritis seemed to correlate with the amount of C1q present in the glomeruli [12]. In addition, other studies have found that the presence of anti-C1q antibodies was associated positively with glomerular C1q deposition [21].

In our study, we confirmed that the presence of anti-C1q autoantibodies is associated with the intensity of glomerular deposition of C1q, both of which were associated with disease activity of SLE and lupus nephritis. In addition, the intensity of glomerular deposition of C1q was also associated with IgG and was co-localized with IgG in the glomeruli. The co-localization indicated that C1q could deposit on the kidney with immunoglobins, including anti-C1q autoantibodies, which has been previously described by Mannik et al. [22]. This finding is strengthened by the findings of Daha et al. who demonstrated that anti-C1q autoantibodies may induce renal disease by deposition in glomeruli only together with C1q and immune complex [12]. Thus, the kidneys are at least one of the target organs of anti-C1q autoantibodies. Though the intensity of vascular deposition of C1q was associated with vascular deposition of IgG and IgM, presence of anti-C1q autoantibodies was not associated with the intensity of vascular deposition of C1q. While a recent study indicated that different immune complex might deposit in glomerular and extraglomerular regions, the pathogenesis of vascular lesions still needs further investigation [12]. Serum levels of C1q were not associated with glomerular deposition of C1q or vascular deposition of C1q. This indicates that serum C1q may contribute little to the deposition of C1q in the kidneys, while local production of C1q by endothelial cells [23,24], dendritic cells, and macrophages in the kidneys might be the main source of the local C1q deposition [25].

Animal studies suggest that immune deposition of C1q and anti-C1q autoantibodies in the kidney is dependent on the presence of glomerular IgG deposition, and additional studies have indicated that anti-C1q autoantibodies deposit in glomeruli, but are only pathogenic in combination with glomerular C1q-containing immune complexes, and deposition of C1q in the glomeruli, such as those that may form in glomerular C1q-containing immune complexes, will induce overt renal disease [12]. Our study supports the theory that auto-antibodies of C1q are co-localized with C1q and other immunoglobins in the glomeruli and might induce glomerular injury in patients of lupus nephritis. The severity was associated positively with the intensity of C1q deposition in the glomeruli. However, the constitution of the immune complex is still unclear. Previous studies indicated that the anti-dsDNA antibody is the best serological biomarker for lupus nephritis. Recently Krishnan et al. have reported that those cross-reacting with glomerular basement membrane components produced immune deposits, [26] which suggested that anti-dsDNA antibodies might be the component of immune complex. Interestingly, recent studies indicated that anti-C-reactive protein (CRP) auto-antibodies and anti-PTX3 autoantibodies also correlated with histopathological activity and disease activity in lupus nephritis [27,28]. As CRP and PTX3 could bind to C1q as reported [29], and local production of CRP and PTX3 in the kidneys were also detected in some studies [30,31]. Thus, the correlations between C1q, CRP, PTX3 and its auto-antibodies in the formation of immune complex in lupus nephritis need further investigation.

## Conclusions

In summary, anti-C1q autoantibodies were closely associated with serum levels of C1q and glomerular deposition of C1q, suggesting the kidney is at least one of the target organs of anti-C1q autoantibodies.

## Competing interests

All authors declare that they have no competing interest.

## Authors’ contributions

Dr. YT carried out the immunoassays, participated in the design of the study, performed the statistical analysis and drafted the manuscript. DS carried out the immunoassays and drafted the manuscript. Dr. L-hW carried out the renal histology studies. Prof. FY, M-hZ participated in the design of the study and revised the manuscript. All authors read and approved the final manuscript.

## Pre-publication history

The pre-publication history for this paper can be accessed here:

http://www.biomedcentral.com/1471-2369/14/63/prepub
